# Animal disease control and prevention—from mechanistic insight to translational innovation

**DOI:** 10.52601/bpr.2026.260901

**Published:** 2026-10-31

**Authors:** Guoliang Zhu, Zixiang Zhu, Haixue Zheng

**Affiliations:** 1 State Key Laboratory of Animal Disease Control and Prevention, College of Veterinary Medicine, Lanzhou University, Lanzhou Veterinary Research Institute, Chinese Academy of Agricultural Sciences, Lanzhou 730000,China

Animal diseases remain a major challenge to animal health, livestock production, food security, and increasingly, public health. Effective control and prevention depend not only on surveillance and intervention, but also on a deeper understanding of pathogen biology, host responses, and the experimental systems that make these questions tractable. The special issue *Animal Disease Control and Prevention* brings together six contributions — three research articles, one protocol, and two reviews — that collectively reflect this continuum from mechanism to methodology, and from discovery to application. Together, they show how structural biology, computational modeling, virology, and functional genomics are converging to address some of the most pressing infectious threats in veterinary medicine.

Two reviews in this issue provide a conceptual framework for understanding virus-host interactions from complementary perspectives. Huang and colleagues examine the diverse strategies by which viruses regulate transcription, comparing pathogens that hijack host RNA polymerase Ⅱ with those that rely on RNA-dependent RNA polymerases or self-encoded transcriptional machinery. By linking genome type, intracellular localization, and immune evasion to transcriptional control, this review offers a unifying view of how viruses reprogram host cells for efficient replication (Huang *et al*. 2026). In parallel, Ling and colleagues discuss lipid droplets as a dynamic battlefield in viral infection. Their review highlights the dual nature of these organelles: viruses exploit them as reservoirs of lipids and energy for replication and assembly, whereas host cells mobilize them as platforms for innate immune defense. This synthesis not only deepens our understanding of virus-host metabolic crosstalk, but also points to lipid metabolism as a promising avenue for antiviral intervention (Ling *et al*. 2026).

Several original studies focus on African swine fever virus (ASFV), underscoring both the urgency and the scientific complexity of this devastating pathogen. Sun and colleagues report a computational-experimental strategy that identifies K-7174 as a promising inhibitor of ASFV RNA polymerase. By combining high-resolution structural information, virtual screening, and cellular validation, the study delivers both a candidate antiviral compound and a practical framework for polymerase-targeted drug discovery (Sun *et al*. 2026). Chen and colleagues, in turn, integrate HDOCK-based docking, AlphaFold3 modeling, and biochemical validation to identify the host protein FAM105A as a potential interactor of the viral ubiquitin-conjugating enzyme I215L. Beyond the specific interaction uncovered, this work illustrates the growing value of combining artificial intelligence-based structural prediction with experimental validation to map virus-host protein interaction networks (Chen *et al*. 2026).

The remaining contributions extend the issue from mechanism to platform and resource development. Tian and colleagues establish a dual-reporter Nipah virus pseudovirus system based on a VSV-ΔG backbone co-expressing GFP and NanoLuc. This platform provides a versatile and accessible tool for studying viral entry, cell tropism, neutralization, and antiviral screening under BSL-2 conditions, thereby lowering a major practical barrier in henipavirus research (Tian *et al*. 2026). In the bacterial field, Guo and colleagues construct saturated transposon mutant libraries for *Brucella abortus* A19 and S2308 and define large sets of putative essential genes through Tn-seq analysis. Their work supplies an important functional genomic resource for dissecting *Brucella* biology and for prioritizing targets relevant to antimicrobial development and vaccine research (Guo *et al*. 2026).

Taken together, the articles collected in this special issue demonstrate that progress in animal disease control increasingly depends on interdisciplinary convergence. Mechanistic insight is being sharpened by structural and computational approaches; experimental bottlenecks are being overcome by safer and more informative model systems; and genome-scale resources are opening new routes toward target discovery and rational intervention. We hope this collection will not only inform readers of recent advances in animal infectious disease research, but also stimulate further innovation in the development of vaccines, antivirals, and other control strategies for important animal pathogens.
